# First person – Ana Cecilia Aliaga Fandino

**DOI:** 10.1242/bio.042556

**Published:** 2019-03-15

## Abstract

First Person is a series of interviews with the first authors of a selection of papers published in Biology Open, helping early-career researchers promote themselves alongside their papers. Ana Cecilia Aliaga Fandino is first author on ‘Reprogramming of the cambium regulators during adventitious root development upon wounding of storage tap roots in radish (*Raphanus sativus* L.)’, published in BIO. Ana conducted the research described in this article while a Master's student in Ji-Young Lee's lab at Seoul National University, Republic of Korea. She is now a PhD student in the lab of Christian Hardtke at the University of Lausanne, Switzerland, investigating developmental genetics in the root vascular system.


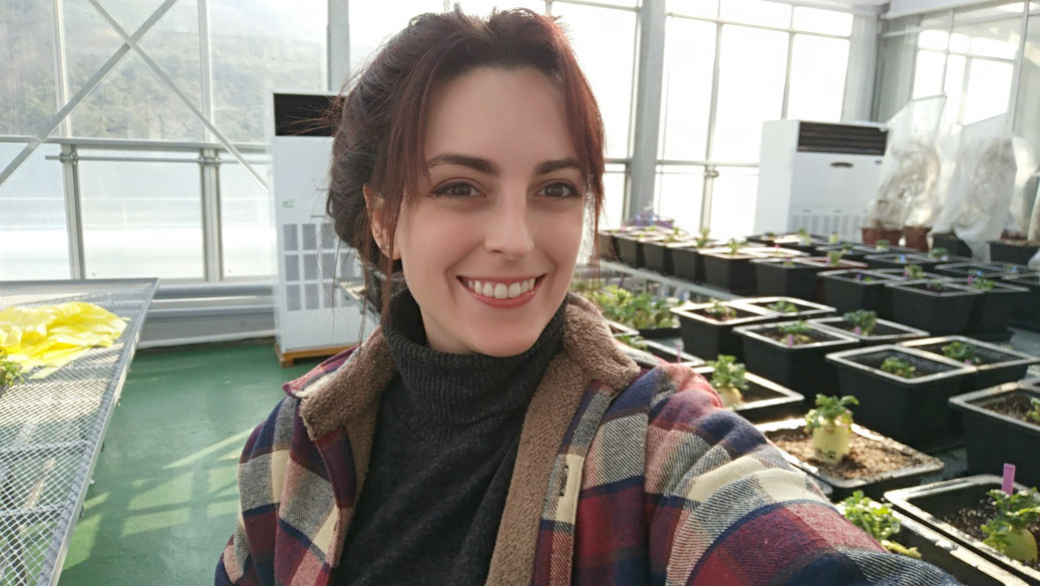


**Ana Cecilia Aliaga Fandino**

**What is your scientific background and the general focus of your lab?**

I have a degree in Biotechnology with a background in plant molecular genetics and business management that I gained during my undergraduate course at Universidad Argentina de la Empresa (UADE). Later, I started my Master's at Seoul National University. Here I was introduced to the molecular genetic studies of vascular development in roots, especially in the radish, a root crop. In the lab I focused on understanding the gene regulatory programs that drive the cell proliferation and cell fate determination in the root vascular stem cells.

**How would you explain the main findings of your paper to non-scientific family and friends?**

Plants have two types of stem cells, the ones that increase the length of a plant and the others that increase the plant's girth. In storage tap roots like radish, carrot and sugar beet, increase in the girth of a root is achieved by the cell division of stem cells called cambium. Cambium cells divide in the lateral direction and generate daughter cells that differentiate into sugar-transporting phloem and water-transporting xylem cells. When this root is accidently severed, the cambium stops cell division in the lateral direction and converts itself into a source of new roots. For this to happen, we found some of genes that normally help lateral cell division in the cambium are reprogrammed to induce the generation of new roots so that the plant can survive. This research shows an exemplary case of recycling gene regulatory pathways to enhance developmental plasticity of plants in order to survive.

**What are the potential implications of these results for your field of research?**

These results open a new field of study on how genes that are known to be used for a specific purpose can reprogram their expression pattern and roles to enhance plant survivorship. Because of the evolutionary proximity of the radish and Arabidopsis, we proposed the use of radish as a model root crop. I find the radish root is a great system for visualizing tissue organization and observing dynamic changes of cambium during the storage root development, which cannot be replicated in Arabidopsis.

**What has surprised you the most while conducting your research?**

During my research I was actually working on the transcriptome of the radish cambium and the study of the genes involved in the radish girth growth, also known as secondary growth. After cutting the radishes in half for this experiment, I replanted them, trying to make them survive for seed production. My biggest surprise was seeing the full recovery of the plant after being replanted, and then seeing the adventitious roots that had formed over the cambium. This surprise drove me to investigate cambium reprograming from secondary growth to adventitious root formation.

**What, in your opinion, are some of the greatest achievements in your field and how has this influenced your research?**

The greatest achievements in my field are the revelation of hormonal crosstalk and their precise balance during plant organ formation and growth. Plant hormones, such as auxin, control the expression of many genes that are key for the plant development and for this process, the location and concentration of the hormone is crucial. And, for example, the change of the auxin concentration during wounding may change the fate of the cambium stem cells, from increasing girth to producing a new root. This was a key point in my own research.

**What changes do you think could improve the professional lives of early-career scientists?**

Depending on the university, Master's and PhD students are sometimes required to do full time research work, combined with teaching hours. In many cases, the mentoring received in this period is partial and the student is required to work independently. Under these conditions, it would be nice if graduate students, that often times have working contracts as researchers, would receive benefits associated with being a full-time worker. This would include a pension, health insurance, and a salary according to the years worked. Another issue that I think needs some improvement is the female researcher's family planning. Many times, women that decide to have a family are seen as unprofessional in the academic environment and they struggle to get positions. An academic researcher has lots of years of work, and sometimes it overlaps with the female biological reproductive period. Thus, it is challenging as a female scientist to plan a family without sabotaging their academic career. It would be important to implement more family-friendly policies that allow researchers the freedom to decide when to have a family without putting their career at risk.

**Figure:**
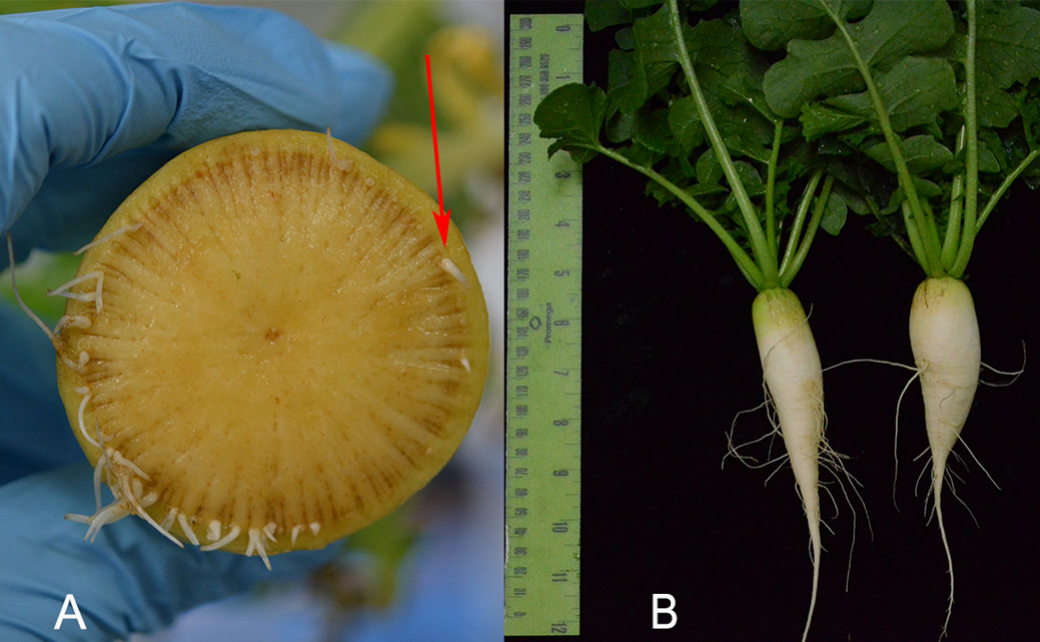
The adventitious roots were formed after cutting the radish in half and growing it 2 weeks in hydroponic media. (A) Shows a radish root creating adventitious roots from the cambium, indicated by a red arrow. (B) Radishes used for the study before being cut.

**What's next for you?**

At the moment I am continuing my academic career as a PhD student at Prof. Christian Hardtke's lab at the University of Lausanne in Switzerland. I want to keep studying the molecular pathways involved in the different elements of the root vasculature system development and their hormonal regulators.

“Brilliant minds are found worldwide but chances are not given equally.”

**Would you like to add something else?**

I would like to take this opportunity to raise awareness of the struggling research community in Argentina. Increasing inflation, the fall of the local currency and bad political policies are currently taking Argentinian research to the verge of collapse. Brilliant minds are found worldwide but chances are not given equally. I hope the scientific community supports each other in this time of need and walks together towards a brighter future for all.
